# The Emergence of Monkeypox: A Global Health Threat

**DOI:** 10.7759/cureus.29304

**Published:** 2022-09-18

**Authors:** Ranjit Sah, Aroop Mohanty, Vivek Hada, Parul Singh, Aishwarya Govindaswamy, Abdelmonem Siddiq, Abdullah Reda, Kuldeep Dhama

**Affiliations:** 1 Medicine (Division of Infectious Disease), Medanta – The Medicity, Gurgaon, IND; 2 Microbiology, Institute of Medicine, Tribhuvan University, Kathmandu, NPL; 3 Clinical Microbiology, All India Institute of Medical Sciences, Gorakhpur, Gorakhpur, IND; 4 Microbiology, Apollo Proton Cancer Centre, Chennai, IND; 5 Pharmacy, Mansoura University, Mansoura, EGY; 6 Neurological Surgery, Faculty of Medicine, Al-Azhar University, Cairo, EGY; 7 Pathology, Indian Council of Agricultural Research (ICAR)Indian Veterinary Research Institute, Bareilly, IND

**Keywords:** sexual route of transmission, public health emergency of international concern, democratic republic of the congo (dcr), smallpox vaccine, lymphadenopathy, orthopoxvirus family, zoonotic disease, vaccination, monkeypox

## Abstract

Monkeypox (MPX) is a zoonotic disease caused by the monkeypox virus (MPXV) belonging to the *Orthopoxvirus* genus. It results in a smallpox-like disease in humans. Recently, MPX has been declared a public health emergency of international concern. The disease is characterized by fever, muscle ache, malaise, and pustules. The presence of characteristic significant lymphadenopathy helps it to be differentiated from other similar illnesses. Early detection of cases and effective contact tracing is necessary for breaking the chain of transmission. Diagnosis can be confirmed by polymerase chain reaction (PCR) testing of the lesions or by demonstrating the virus in other body fluids. There is no specific treatment for monkeypox, although the smallpox vaccine is thought to have high levels of protection. In this review, we have tried to collect all relevant information about the current outbreak, including epidemiological data, modalities of diagnosis, and treatment options

## Introduction and background

Human monkeypox (MPX) is a zoonotic viral disease first detected in parts of North Africa in the 1970s. It is caused by a monkeypox virus (MPXV), a double-stranded DNA virus, belonging to the genus *Orthopoxvirus *of the family *Poxviridae* [1]. It is closely related to the long-eradicated smallpox caused by the variola virus [2]. Moreover, it results in a smallpox-like disease. Small rodents are considered to be the natural reservoir for infections caused by this virus; however, animal reservoirs are yet to be confirmed. It has been evidenced that humans and monkeys are occasional hosts of the virus. The name monkeypox originated with the isolation and identification of the virus in captive cynomolgus monkeys (*Macaca fascicularis*) at a laboratory in Denmark in 1958 [3,4]. However, it took 12 years for the virus to pass to humans when the first human case of MPX was reported from a paediatric patient in the Democratic Republic of the Congo (DCR) in 1970 [5,6]. Since then, the disease remained restricted in various countries of Central and West Africa until 2003, when a major outbreak was seen in the United States of America (USA) following the importation of animals [7].

Over the last two decades, reports indicated sporadic cases of MPX from North America, Europe, and the Middle East, mostly linked to travellers to those countries. One possible reason for the remarkable increase in cases could be the reduced frequency of vaccinating against smallpox, which provided some cross-protection against MPXV [8]. Recent reports showed a multi-country outbreak of MPXV in May 2022, affecting several continents, including non-endemic and endemic countries. Surprisingly, most confirmed cases reported a travel history to North America or Europe, rather than African countries where the disease is endemic. Most of the cases were males aged between 20-49 years and men having sexual intercourse with men (MSM) [9]. Very recently, on July 23, 2022, the WHO declared MPX as a public health emergency of international concern, and presently more than 21,000 cases have been reported from over 75 countries as of July 28, 2022 [10].

MPXV can significantly spread to humans from rodent bites and close contact with bodily fluids of infected live or dead animals. Human-to-human transmission might also occur by respiratory droplets, contact with infected lesions, or bodily fluids. Besides, it has been demonstrated that MPXV can be detected in genital and rectal lesions and seminal fluid in confirmed cases in Italy [11]. This further reemphasizes the sexual route of transmission observed in most worldwide patients. The incubation period of the disease ranges from 5-15 days [10]. The infection undergoes two phases within its completion, including the invasion and cutaneous phases. In the initial period, it is very difficult to distinguish the disease from smallpox, chickenpox, and measles. The cutaneous phase is characterized by a rash involving the extremities and pustules on the oral mucous membranes and genitalia, along with lymphadenopathy, which differentiates it from other similar infections. We have presented the differences between these illnesses in Table 1.

**Table 1:** Comparison of smallpox, chickenpox, and monkeypox dsDNA : double-stranded deoxyribonucleic acid

Characteristics	Monkeypox	Smallpox	Chickenpox
Causative agent	Monkeypox virus, (dsDNA virus, Family: *Poxviridae*)	Variola virus, (dsDNA , Family: *Poxviridae* )	Varicella-zoster virus (dsDNA, Family: *Poxviridae*)
Animal reservoir	Yes	No	No
Mode of transmission	Contact with exotic animals in endemic regions; contact with infected humans	Droplet	Droplet
Asymptomatic infections			
Reactivation	No	No	Yes (herpes zoster)
Protection with smallpox vaccine	Partial	Yes	No
Vaccine availability	Yes	Yes	Yes
Incubation period	5-21 days	10-14 days	14-16 days
Prodromal fever	Yes	Yes	Yes
Lymphadenopathy	Yes (submandibular, cervical, and sublingual region)	No	No
Involvement of body	Centrifugal (80%) or centripetal (20%)	Centrifugal	Centripetal
Depth of lesions	Superficial	Deep	Superficial
Evolution of lesions	Monomorphic (80%), pleomorphic (20%)	Monomorphic	Pleomorphic
Involvement of palm	Yes	Yes	No

Given the rapid spread of the virus and its impact on global health, the research community responded with many preliminary research articles about this multi-country outbreak. Here we have tried to summarize all the available important literature about this deadly re-emerging virus covering all relevant information regarding MPXV.

## Review

Monkeypox is an enzootic infection, primarily seen in small rodents/mammals of Central and Western Africa. The causative agent was first isolated in a captive monkey in Denmark, but the virus is naturally transmitted among small rodents and mammals in African countries. Evidence shows that squirrels (especially *Funisciurusanerythrus*) inhabiting agricultural areas in the DRC are considered primary candidates to sustain viral transmission among people in nearby settlements [12].

**Figure 1:**
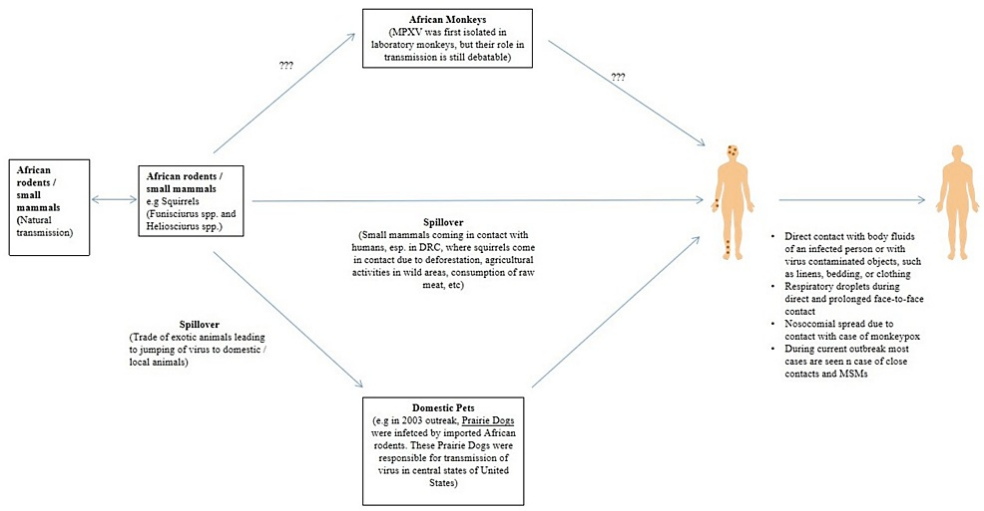
Transmission of Monkeypox DRC: Democratic Republic of Congo; MPXV: monkeypox virus; MSM: men who have sex with men Image: original illustration

Deforestation, using forest land for agriculture, leads to the interaction of these animals with humans and increases the chances of transmission. Moreover, exporting exotic animals to non-endemic areas can lead to viral transmission to other animals and subsequently to humans. Having direct contact with virally contaminated objects, like clothing, bedding, or linens, might spread the infection. Respiratory droplets are mainly responsible for person-to-person transmission, usually during prolonged direct face-to-face contact. Direct contact with bodily fluids of infected patients might also spread the infection (Figure 1) [2].

Monkeypox and the African continent

Historical evidence shows that the first human MPX was recorded in 1970 from the DRC [6]. Since then, different reports have indicated viral transmission in the rainy forests of small villages located in Central and Western Africa. The DRC continues to be the most impacted by MPX. During the past five decades, continuous MPX cases were not reported in countries other than the DRC. Moreover, thousands and hundreds of suspected cases and deaths of MPX have been reported in the DRD within the past decade. The second most affected country is Nigeria, which had an outbreak in September 2017, during which 181 confirmed and probable cases were detected [13]. In Central Africa, an estimated mortality rate of 10.6% was reported, attributed to a more virulent MPX strain causing the infection in this region[14]**.**

First outbreak outside Africa

In 2003, importing wild/exotic animals from Africa led to the first appearance of MPX outside Africa, in USA. For instance, many small African rodents/mammals, like striped mice (*Hybomys* spp), dormice (*Graphiurus* spp), Brushtail porcupines (*Atherurus *spp), Gambian pouched rat (*Cricetomys gambianus*), sun squirrels (Heliosciurus spp), and rope squirrels (*Funisciurus* spp), were imported from Ghana [15]. Some of these animals were infected with MPXV, and being kept proximal to prairie dogs before their distribution led to a viral transmission. The supply of these prairie dogs led to the spread of the virus in most cases. Moreover, rabbits in close contact with an ill prairie dog caught the infection and were considered the main source of transmitting the illness to one person. However, it has been shown that the virus could not be isolated from the rabbit. During this outbreak, a total of 81 cases were identified (40% were laboratory confirmed) [7,16].

The smallpox vaccine was used to contain the spread of MPX [7]. Moreover, importing African rodents and mammals, like the striped mice (*Lemniscomys* spp), dormice, brush-tailed porcupines), African giant pouched rats, sun squirrels, and rope squirrels were banned in the USA during this period to reduce viral transmission. No MPX-related deaths were reported during the outbreak, and no person-to-person transmission was recorded. According to a previous investigation that was conducted during the outbreak, MPX was associated with specific activities related to animals, like being bitten, scratched, or bitten by an infected animal through broken skin. Besides, it was observed being in contact with the bedding or cleaning a cage of infected animals are other essential factors that can help disseminate the viral infection and spread of the disease [17].

Sporadic cases outside Africa until January 2022

After 2003, there had been no significant outbreaks of MPX outside Africa. Most cases were attributed to travelling to African countries. In September 2018, two epidemiologically unlinked cases were detected in the United Kingdom. Both patients had a history of travelling to Nigeria prior to the onset of the illness [18]. During the hospital stay, a healthcare worker also caught the infection secondary to contact with blood/body fluids of one of the infected cases [19]. During the same period, a case was identified in Israel in a man who returned from Nigeria and developed an erythematous rash. He had disposed of rodent carcasses during his visit [20].

In May 2019, a case was identified in Singapore in a person who travelled to Nigeria. In this case, there was neither a history of contact with rodents/wild animals nor individuals with the pox-like illness. However, ingestion of potentially contaminated barbecued bushmeat was reported, which might be the reason for catching the infection [21]. It is noteworthy that in 2017 there was a re-emergence of MPX in Nigeria, and there was a large outbreak, accounting for more than 200 cases. Subsequently, travel to Nigeria was associated with many cases outside Africa during this period [22]. In London, three cases were detected in 2021 after the coronavirus disease 2019 (COVID-19) lockdown among family members returning from Nigeria. During that episode, the number of cases did not remarkably increase, owing to mandatory post-travelling isolation of travellers and the use of personal protective equipment (PPE) by healthcare workers [23].

Current outbreak outside endemic regions

The WHO reported the current first MPX case on May 7, 2022. It was seen in an individual who had a travel history to Nigeria in April, and while returning, he developed a vesicular lesion. In this case, reverse transcription-polymerase chain reaction (RT-PCR) was used to confirm the diagnosis. Following this, immediate contact tracing was initiated, and the patient was kept isolated [23]. Within one week of this event, some reports indicated the detection of two confirmed and one suspected MPX case within the same household. Moreover, it was further estimated that a vesicular rash illness was reported in four more laboratory-confirmed cases in individuals identified as MSM, according to the Sexual Health Services [23]. In these seven cases, there was neither a history of travelling to Africa nor any contact with exotic animals. These infections appeared to have been locally acquired in the United Kingdom [24].

Within a few days after the detection of these cases, the number of cases significantly increased in multiple countries. Most cases were reported in four WHO regions, including the Western Pacific, European, Eastern Mediterranean, and the Americas. The WHO declared monkeypox a public health emergency of international concern owing to continuously rising cases with more than 21,000 cases recorded currently from more than 75 countries as of July 28, 2022 [10].

During this outbreak, most cases were observed in males aged 18-44. Besides, in subjects with sexual orientation, more than half were identified as homosexual, bisexual, and other men identified as MSM [25]. As of July 25, 2022, 173 confirmed cases had been reported in Africa, together with three deaths [24]. On the other hand, no deaths were reported outside the African region.

Laboratory diagnosis

MPX diagnosis confirmation mainly depends on the quality of the collected samples, specimen type, and the type of test performed in the laboratory. Specimens must be collected appropriately and require safe transportation to the laboratory with adequate infrastructure to perform the tests [26]. The diagnostic samples should include the skin lesions, which can differ based on the phase of the rash. Specimens should consist of vesicle fluid, pustules, or dry crusts from the healing lesion and nasopharyngeal or oropharyngeal swabs in a dry tube without any viral transport medium. If possible, biopsy specimens might also be obtained. Blood in ethylenediamine tetraacetic acid (EDTA)/serum-separating tubes (SSGT) and urine in a sterile container can also be collected during the recovery phase [27]. Personal protective equipment (PPE) like gowns, N95 masks, goggles, and face shields should be worn by the healthcare professional while collecting specimens [28].

Two swabs from each lesion should be collected, preferably from various areas of the body, and should include lesions that appear different. Any synthetic swabs made of Dacron, polyester, or nylon can be used. Cotton swabs should be avoided. The lesion has to be swabbed vigorously for DNA collection. The swab has to be transferred to a leak-proof sterile tube with a gasket seal. Specimens have to be kept and transported to the laboratory within one hour under refrigerator temperature (2-8°C) or can be frozen under -20°C [29]. In setups where a cold chain facility is not available, the stability of viral DNA can be maintained intact by keeping specimens in a dark and cool place.

The direct MPXV identification methods can be followed by isolating the virus from the clinical specimens using electron microscopy and immunohistochemistry to detect viral antigens. Viral culturing can also be done in chorioallantoic membranes (CAM) which produce pock-like lesions, or in vitro cell lines of green monkey kidneys where cytopathic effects (CPE) like detachment of cells and rounding can be seen [30]. In electron microscopy, the virus appears as brick-shaped particles with lateral bodies and a central core measuring around 200-300 nm. However, this method cannot differentiate other viruses of the Orthopox family [31]. The above diagnostic methodologies require experienced technical staff and reference laboratories with containment facilities and, thus, are not recommended for routine diagnostics. 

Serology for detecting IgM and IgG antibodies is limited due to cross-reactivity to other orthopoxviruses' antibodies and requiring cold chain facilities. IgM levels indicate recent exposure to orthopoxvirusesor getting a smallpox vaccination. On the other hand, the presence of IgG antibodies indicates past exposure to orthopoxviruses either by natural infection or vaccination. Therefore, the presence of both IgM and IgG strongly suggests recent exposure to orthopoxviruses in individuals who had natural infection or vaccination [32]. The first point-of-care test to detect *Orthopoxvirus* spp is the Orthopox BioThreat Alert® (Tetracore, Inc., Rockville, Maryland, USA), based on the principle of lateral flow assay, developed in 2003. It can be done at ambient temperature with not much expertise. However, it is not specific to MPXV and can only be used as a screening test [33].

Molecular methods include PCR, RT-PCR, and restriction fragment length polymorphism (RFLP) and are usually performed in a facility with Biosafety level 3 (BSL-3). Unlike other diagnostic methods, molecular ones are highly sensitive and have a high throughput. Many communities have designed standardized PCR protocols for detecting orthopoxvirus. They are even more specific to detecting MPXV and can differentiate the West African and Congo Basin clades. The RT-PCR assays usually target conserved regions, like the *F3L* gene, the DNA-dependent RNA polymerase subunit 18 (rpo18), the envelope protein gene (*B6R*), and the DNA polymerase gene (*E9L*). Moreover, the RFLP of the amplified gene products can be used to detect MPX viral DNA. However, it requires culturing the virus and is a time-consuming process. Therefore, its use is not recommended in clinical settings. Whole genome sequencing, like next-generation sequencing (NGS), is the current gold standard for characterizing different viruses of the *Orthopoxvirus* genus, including MPXV. However, the technology is highly expensive, requires expertise and knowledge to analyse the sequenced data, and cannot be used in poor and resource-limited settings [32,34].

Treatment and prevention

No specific treatment approaches have been proposed for MPX. However, evidence shows that the main line of treatment includes symptomatic management, supportive care, and managing secondary events, like bacterial infections. Moreover, specific measures should be considered for these factors developed and evaluated in different care settings, using clinical measures for MPX combined with antiviral treatment. Various compounds showing promising results for antiviral therapies against *Orthopoxvirus* species include CMX001 (brincidofovir, sold under the brand name Tembexa) and ST-246 (tecovirimat, sold under the brand name TPOXX) [35-37]. These compounds are summarized in Table 2. These antiviral drugs are used in various combinations with intravenous administration of vaccinia immune globulin (IV-VIG) to treat severe vaccine-associated adverse events [38,39].

**Table 2:** A summary of antiviral therapeutics proposed for monkeypox infection.

Antiviral Therapeutic	Mechanism of Action	Clinical Considerations	Stage of Development or Use	Adverse Reactions	Efficacy
Cidofovir (Vistide): It has antiviral activity against a variety of viruses by inhibiting viral DNA polymerase.	Inhibits DNA polymerase.	Intravenous administration with hydration and probenecid	Licensed for the use of cytomegalovirus retinitis in AIDS patients, has been used to treat other poxvirus infections (molluscum contagiosum and Orf virus (sore mouth infection)).	Nephrotoxicity has been seen.	It has been shown to be effective against orthopoxviruses in in-vitro and animal studies.
CMX-001/Brincidafovir (TEMBEXA^®):^ It is a modified cidofovir compound that lacks the extent of nephrotoxicity seen with cidofovir. Antiviral activity of CMX-001 has been demonstrated with a variety of *Orthopoxvirus* species.	Modified cidofovir compound; inhibits DNA polymerase.	Lacks nephrotoxicity seen with cidofovir; oral administration.	In development.	Nausea, vomiting, and abdominal pain	It has been shown to be effective against orthopoxviruses in in-vitro and animal studies. Efficacy can be reduced in immunocompromised patients based on studies on immune-deficient animals.
ST-246/tecovirimat (TPOXX): The drug ST-246 blocks the release of the intracellular virus from the cell and has shown promising results against a variety of *Orthopoxvirus* species, including variola virus	Inhibits release of intracellular virus	Oral administration (200 mg)/ Intravenous administration Drug absorption of oral formulation is dependent on adequate intake of full, fatty meals. IV tecovirimat should be contraindicated in patients with renal impairment (CrCl<30ml/min)	It is maintained in the United States in the Strategic National Stockpile, available for other orthopoxvirus infections under an investigational protocol Treating considerations: With severe disease (sepsis, encephalitis, confluent lesions, etc.) Who are at high risk of severe disease (immunocompromised persons, paediatric population, pregnant and nursing mothers, having secondary bacterial infections) With atypical infections involving accidental implantation in eyes or mouth or other anatomic areas).	Headache, nausea, abdominal pain, vomiting, Neutropenia (rarely), Infusion site: pain, erythema, swelling, extravasation	Shorten the duration of illness and virus shedding [36]

Healthcare authorities in endemic areas should adopt and create different strategies to formulate drugs that can specifically treat the MPX disease. Preventing MPXV transmission in endemic areas is critical. It can be done by different approaches, including vaccination, avoiding contact with rodents and primates, including their bodily fluids, and not eating the undercooked meat of infected animals. Proper handling of potential animal reservoir species by wearing PPE (including surgical masks, gowns, and gloves) is recommended. Avoiding contact with infected patients is also suggested to reduce human-to-human transmission. Efforts should also be directed to implement infection control strategies to reduce person-to-person transmission, especially among healthcare workers. Healthcare professionals are encouraged to follow standard precautionary practices and isolate themselves whenever an infection is suspected. Adopting health education campaigns to increase public awareness about the disease should also be encouraged.

It has been estimated that smallpox vaccination provides 85% cross-protection against MPXV infection. Healthcare officials should consider ring immunization against smallpox for healthcare professionals and those treating or exposed to patients with MPX [2]. Smallpox vaccination is recommended within 14 days (ideally within four days) after significant exposure to a diseased animal or a confirmed human case, according to the Centers for Disease Control and Prevention (CDC) [28]. Isolation of infected persons and contact tracing for six weeks since the exposure has been a recommended prevention practice amid the MPX outbreak. Compliance with specific directives of public health authorities is compulsory. Furthermore, creating awareness for sampling, surveillance, and continual education alongside action both by local and global authorities is of prime importance.

In a suspected case of monkeypox, when a patient has a history of fever, dermal lesions, close contact, or travel to an endemic area, it is recommended to immediately place the patient in a negative air pressure isolation room, or an isolated room if such facilities are not available. Compliance with standard, contact, and droplet precautions is to be observed among healthcare workers. The infection control team should be immediately informed. Increasing awareness among healthcare workers about the disease and its endemic areas should be focused on [40,41].

Environmental survival and disinfection

It is suggested that standard cleaning and disinfection procedures using an Environmental Protection Agency (EPA)-registered hospital-grade disinfectant with an emerging viral pathogen might be effective. However, orthopoxviruses are known to be hardy and can survive on environmental surfaces for weeks or months. The virus can harbour for a longer time in porous materials like bedding, clothing, etc., than on non-porous (plastic, glass, metal) surfaces. Besides, the orthopoxviruses are very sensitive to light and also sensitive to disinfectants like hypochlorous acid, isopropanol, quaternary ammonium, and hydrogen peroxide [28].

Post-exposure prophylaxis

According to the CDC, for patients with high/intermediate risk, receiving a vaccine within four days of exposure prevents the onset of the disease. On the other hand, while vaccines given 4-14 days after exposure may reduce the symptoms of MPX, they may not prevent the disease. Moreover, it has been recommended that post-exposure prophylaxis (PEP)++ (individual-directed PEP, expanded PEP, or PEP plus-plus) should be practised by people with certain risk factors (with a high probability of exposure) even if they have not had documented exposure to confirmed MPX cases. Besides, these practices should be encouraged in areas with high MPXV transmission (outbreak response) [42].

Vaccines against monkeypox

According to official reports, no currently available vaccines are specific to preventing MPX. The current vaccines that might prevent MPXV infection should be evaluated by controlled clinical trials to look for the impact of using smallpox vaccines for the prevention and altering the severity of the disease. CDC recently recommends pre-exposure smallpox vaccination for healthcare workers, field investigators, veterinarians, and contacts with MPX patients. Besides, populations with unknown immunocompromised profiles pose serious concerns as smallpox vaccines, composed of fully replicative vaccinia virus, are currently not in use in the MPX endemic areas due to serious adverse events [43].

Vaccinia virus-based vaccination represents a hybrid of cowpox with other orthopoxviruses. CAM is the cell culture mostly used to identify and cultivate viruses [44,45]. The vaccinia virus strain administered most often for smallpox vaccination was the Lister strain; New York City Board of Health (NYCBOH) strain (Dryvax, Wyeth, LLC, Madison, New Jersey, USA, now defunct). This was prepared from calf lymph (Acambis & Baxter Laboratories, UK: ACAM2000, German Research group: MVA) by serial 500 passages in Chicken Embryo Fibroblasts (CEF). This showed 15% genome deletion. Other strains which have been used are EM-63 in the Soviet Union, Temple of Heaven Strain in China, and Patwadanger strain in India. It was chosen to make recombinant vaccinia virus intended for human use [46-48].

Replicative vaccines such as ACAM2000 can balance adverse events from a pathogenic MPX disease [49,50]. Vaccines are considered ideal for use in MPX-endemic areas and are readily available to be administered in children with lesser adverse effects and groups with a reduced risk profile. Modified vaccinia Ankara (MVA) is an attenuated vaccinia virus. It should be noted that the replication in mammalian cells is very limited in this virus. With lethal doses of MPXV, MVA has shown an enhanced safety profile in primate models. However, in primates with severely diminished T-cell functions, this vaccine has not shown protection [51]. Furthermore, LC16m8 is another vaccine that has shown protection against severe monkeypox illness in non-human primates as it has been altered to prevent viral replication. It has been reported that LC16m8 has reported very few adverse events after vaccinating more than 50,000 school children in Japan [52]. A summary of all available vaccines is presented in Table 3.

**Table 3:** Currently available vaccines for monkeypox infection. PEP: post-exposure prophylaxis; PrEP: pre-exposure prophylaxis

Vaccine	Dose Administration	Contraindications	Efficacy	Adverse Reactions	Stage of Development or Use
ACAM2000 (Acambis, Inc., Cambridge, Massachusetts, United States): Live vaccinia virus	Single-dose administration	Immunocompromised conditions (HIV), cardiac disease, eye disease treated with topical steroids, atopic dermatitis/eczema, and persons with a history of atopic dermatitis/eczema or other acute or exfoliative skin conditions, infants less than 12 months of age, pregnancy	No data for PEP and PrEP from the current outbreak	Injection site pain, swelling, and redness; fever; rash; lymph node swelling; and complications from inadvertent inoculation	FDA approval for ACAM2000 in 2007; licensed vaccination in the United States.
Modified vaccinia Ankara; IMVAMUNE (US); JYNNEOS; IMVANEX (Europe): Attenuated vaccinia virus	Two-dose administration	Serious vaccine component allergy	No Data for PEP and PrEP from the current outbreak	Injection site reactions such as pain, swelling, and redness	Licensed by FDA in September 2019 for prevention of small-pox and monkeypox in adults (>18 yrs) November 2021, ACIP: JYNNEOS as an alternative to ACAM2000 for primary vaccination and booster doses as PrEP (occupational risk)
LC16m8: Attenuated vaccinia virus	Single-dose administration		Shown protective efficacy in animal studies	Injection site reactions were mild Mostly pruritis seen.	Licensed for use in Japan

## Conclusions

Monkeypox has been considered a neglected disease during the past few decades; however, its increasing outbreaks seen in the last few years, and the currently rising cases in multiple countries, especially in non-endemic regions beyond Africa, have now placed it a disease of public health emergency of international concern. After the eradication of smallpox many a year back, smallpox vaccination was stopped which has resulted in a larger population (aged below 40-50 years) having lesser protection against MPX. In-depth research investigations, wider epidemiological and molecular studies, rapid and confirmatory diagnosis, finding natural animal reservoirs, understanding associated zoonosis in a better way, adopting adequate prevention and control measures, and developing effective and specific vaccines, drugs, and therapies require a global effort of worldwide researchers, public health expert,s and health agencies. Moreover, vaccines and antiviral drugs need to be made widely available as presently they are of very limited global access. Rising cases of MPX need to be halted timely by designing appropriate proactive control and implementing adequate preventive measures as well as formulating future preparedness plans effectively so as to avoid any MPX pandemic amid the already ongoing COVID-19 pandemic. 
